# Bacteriological profile, risk factors and antimicrobial susceptibility patterns of symptomatic urinary tract infection among students of Mekelle University, northern Ethiopia

**DOI:** 10.1186/s12879-019-4610-2

**Published:** 2019-11-08

**Authors:** Guesh Gebremariam, Haftom Legese, Yemane Woldu, Tadele Araya, Kiflom Hagos, Araya GebreyesusWasihun

**Affiliations:** 1 Department of Medical Laboratory, College of Medicine and Health Science, Adigrat University, P.O. Box, 50, Adigrat, Ethiopia; 2 Department of Microbiology and Immunology, Institute of Biomedical Sciences, College of Health Science, Mekelle University, P.O. Box, 1870, Mekelle, Ethiopia

**Keywords:** Urinary tract infection, Antibiotic susceptibility, Bacteriological profile, Ethiopia, Mekelle University

## Abstract

**Background:**

Bacterial infection of the urinary tract is among the common reasons for seeking medical attention in the community. Rapidly increasing antibiotic resistance of uropathogens is resulting in limited treatment options. Therefore, knowledge of the current uropathogens and their antibiotic susceptibility is important for better treatment of urinary tract infection.

**Methods:**

A cross-sectional study design was conducted from February to September thirty, 2017 among students who came to Mekelle University student’s clinics with symptomatic urinary tract infection during the study period.. Mid-stream urine specimens were collected from 341individuals with suspected urinary tract infection for bacteriological identification and antimicrobial susceptibility testing. Data on socio-demographic, clinical and risk factors were also collected using a structured questionnaire.

**Results:**

Among the 341 study participants, 72(21.1%) showed significant bacteriuria. *Escherichia coli* (48.6%), Coagulase-negative staphylococci (23%), *Staphylococcus aureus* (13.5%), and *Klebsiella* spp*.* (8.1%) were common bacterial isolates. Resistance to ampicillin (81–100%), amoxicillin/clavulanic acid (77–93.6%), co- trimoxazole (55 72.3%), nalidixic acid (57.4%) and tetracycline (46–55.5%) was seen by most isolates. Multidrug resistance was observed in 73% of the bacterial isolates, and 25.5% of the Gram-negative isolates were extended-spectrum beta-lactamase producers. Being female, a history of urinary tract infection, a history of catheterization and frequent sexual activity were found to be statistically associated with urinary tract infection.

**Conclusion:**

Urinary tract infection is a problem among university students with a prevalence of 21.1%. All isolates have developed resistance to most of the commonly used antibiotics. Therefore, health education on the transmission and causes of urinary tract infection are recommended for the students.

## Background

Urinary Tract Infection (UTI) is the presence of significant bacteria in urine irrespective of the site of infection in the urinary tract [1].UTI can range from the presence of bacteria in urine without symptoms to serious symptomatic illnesses such as urethritis (urethra), cystitis (bladder), urethritis (ureters), and pyelonephritis (kidney) [2, 3]. It is the most common cause of morbidity in the general population and hospital visit [4, 5].

Globally, 150 million people are diagnosed with UTI yearly [6, 7] which causes an expense of greater than 6 billion US dollars healthcare expenditure in treatment and work loss [8].

UTI occurs in all age groups of both genders [4, 9]. This is as a result of anatomic position, physiological changes, vaginal intercourse, use of contraceptive methods like spermicide and diaphragm, and lack of prostatic fluid which acts as an antibacterial agent, almost 50% of women experiencing at least one episode of UTI during their lifetime [1, 10, 11]. Even though the episode of UTI is less in men than females, it is more serious when it happens [12, 13].

Despite these clear increased risks of UTI, clinicians lack scientifically valid methods to identify and ultimately treat patients with UTI complains [14, 15]. Therefore, UTI can cause serious complications such as frequent recurrences, bacteremia, and renal failure, preterm delivery [16, 17].

UTI among university students is commonly similar to the general population and the majority of the females have recurrent infections within 1 year [18]. The prevalence of UTI in India and Saudi Arabia among College students were found to be 19.8 and 32.1% respectively [5, 19]. UTI among university students of Africa (Nigeria) has been reported as 28% [20].

The causative agent of UTI is predominantly associated with Gram-negative bacteria such as *E. coli* (75–90%), *Klebsiella* spp. Proteusspp., and Gram-positive bacteria such as coagulase-negative staph (CoNS) and *S.aureus* [21].

Nowadays, uropathogen isolates are alarmingly exhibited a high percentage of resistance to almost all antibiotics worldwide [22]. This owning to the irrational use of antibiotics among university students [23–26].

In Ethiopia concerning the prevalence and antimicrobial susceptibility pattern of UTIs among hospital patients varies from area to area [7, 27], and there is no published data in Mekelle city as well as in Ethiopia concerning the UTI among University students. Updated information on UTI and its prevalence and resistance patterns are very important for the proper selection and use of antimicrobial agents in a setting. Thus, this study was aimed to assess bacteriological profile, risk factors and antimicrobial susceptibility patterns of symptomatic urinary tract infection among students of Mekelle University, northern Ethiopia.

## Methods

### Study design, period and area

A laboratory-based cross-sectional study was conducted from February to September thirty, 2017 among university students at Mekelle University, Mekelle, northern Ethiopia. Mekelle city is the capital city of Tigray Regional State and found at a distance of 783 km from Addis Ababa which is the capital city of Ethiopia. Its elevation is 2084 m above sea level with an area of 24.4 km^2^. The region has four universities among that one is MekelleUniversity which has five campuses. The names of the campuses are Endayesus main campus, Adi Haqi campus (College of Business and Economics), Ayder campus (College of Health Sciences and Ayder Referral Hospital), Kalamino Campus (College of Veterinary Medicine), Aynalem campus (Mekelle Institute of Technology).

### Sample size determination and sampling technique

The sample size was determined using the single population proportion formula.

$$ \mathrm{n}=\frac{{\mathrm{Z}}^2\upalpha /2\ \mathrm{P}\ \left(1-\mathrm{P}\right)}{{\mathrm{d}}^2} $$

Since there was no data in Ethiopia, the prevalence of UTI among university students was taken from Nigeria (28%) which was done by Nwosu et al [20]. Then with a margin of error (5%), (d = 0.05) and 95% level of confidence (z = 1.96), the sample size was calculated as follows:

$$ \mathrm{n}=\frac{(1.96)^2\ast 0.28\ \left(1-0.28\right)}{(0.05)^2}=310\  plus\ contingency\ 10\% so,310+31=341 $$

Therefore, a total of 341 UTI suspected students were included in the study from all campuses of the university. A convenient sampling technique was used to select study participants from each campus of MU student’s clinic during the study period.

The distribution of the 341 study participants into each campus was done based on proportion to the size of the source population of each campus using proportionate allocation formula.

### Data collection and laboratory processing

Data related to socio-demographic characteristics (gender, age, residence), risk factor associated with UTI (sexual activity, history of UTI,contraceptive use, history of catheterization, history of diabetes mellitus and circumcision [2], and clinical data such as Hematuria/dark Flank pain, urgency frequency,dysuria, abdominal discomfort and urinary incontinence were collected by direct interview of the study participants in combination with a review of medical records. All the questionnaires were checked for accuracy and completeness. After proper instruction, female study participates were informed to clean their peri-urethral area with water and soap then cleanse with sterile gauze to collect 5-10 ml of freshly voided midstream urine using sterile and wide-mouthed plastic bottles with a tight cap. Then the collected midstream urine specimens were transported to Ayder referral hospital medical microbiology lab using (0.1 g) boric acid preservative. Urine specimens were processed in the laboratory within 2 h of collection and specimens that are not processed within 2 h were kept refrigerated at 4 °C until analyzed [28, 29].

### Cultivation and identification isolates

Using calibrated inoculating loop 0.001 mL [2] of uncentrifuged, uniformly mixed, midstream urine samples were aseptically inoculated onto mannitol salt agar (Oxiod, Hampisher, UK), blood agar (Oxiod, Hampshier, UK) and MacConkey agar (Oxiod, Hampshier, UK). After overnight incubation at 37 °C for 24–48 h colonies were counted to check significant growth. Colony counts yielding bacterial growth of 105/mL of urine were regarded as significant for bacteriuria [30]. All positive urine cultures with significant bacteriuria were further identified by their colony characteristics, Gram-stain, and pattern of biochemical profiles using standard procedures. *Enterobacteriaceae* were identified by H_2_S production and carbohydrate utilization tests in TSI agar, motility test, urease test, oxidase, indole test, and citrate tests. The Gram-positive bacteria were identified using catalase and coagulase tests [6, 29].

### Antimicrobial susceptibility testing

Antimicrobial susceptibility test was performed using a modified Kirby- Bauer disc diffusion method according to Clinical and Laboratory Standards Institute (CLSI) guidelines, [31]. The following antibiotics were used: ampicillin (AMP; 10 μg), ceftriaxone (CTR; 30 μg), chloramphenicol (C; 30 μg), amoxicillin/clavulanic acid (AMC; 20/10 μg), erythromycin (ERY; 15 μg), gentamicin (GEN; 10 μg), nalidixic acid (NA; 30 μg), nitrofurantoin (NIT; 300 μg),co-trimozaxole (COT; 25/125 μg), ciprofloxacin (CIP; 5 μg),norfloxacin (Nx; 10 μg) and tetracycline (TE; 30 μg). Isolates were classified as sensitive, intermediate and resistant according to the standardized table supplied by CLSI 2016 [32] .ESBL screening was also performed based on the disk diffusion clavulanate inhibition test using ceftazidime /clavulanic acid, ceftazidime, and cefotaxime (Himedia) antibiotic discs [32].

### Quality control

Strict measures were taken from the pre-analytical to the post-analytical phase. The questionnaire was pretested in 17 patients with symptomatic urinary tract infection at Adigrat university student clinic, Northern Ethiopia. As part of quality assurance, 2 days of training was given for data collectors on how to perform the questionnaire and sampling process. The legibility of the filled questionnaire and any labeling errors were confirmed immediately. Laboratory analyses were carried out using standard operating procedures. Culture media were tested for sterility and performance by incubating 5% of the batch [21]. Standard reference strains of *Staphylococcus aureus* (ATCC25923), *Escherichia coli* (ATCC25922), *P. aeruginosa* (ATCC27853) and *P.mirabilis* (ATCC 25659) were used during culture and antimicrobial susceptibility testing.

### Data processing and analysis

Data were edited, cleaned, entered and analyzed using statistical package for social science (SPSS) version 22. Descriptive statistics, Bivariate, and multivariate logistic regression were performed. Bivariate logistic regression was employed to look association between the outcome variable and each independent variable. A binary logistic regression analysis was used to calculate the odds ratios (OR); Crude Odds Ratio (COR) and Adjusted Odds Ratio (AOR) to ascertain the degree of association between risk factors of symptomatic urinary tract infection. In this study, multi-collinearity among independent variables was detected using the standard errors for regression coefficients. The corresponding variables with *P*-value (*P* < 0.05) and the 95% confidence interval were then considered as statistically significant differences.

## Results

### Socio-demographic characteristics

Out of 341 eligible students with symptoms of urinary tract infection, all are agreed to participate in this study, which made a response rate of 100%. Of whom, 244(71.6%) were females and 97(28.4%) were males with a 1:2.5 male to female ratio. The majority of the study participants 217(63.6%) were in the age group of 21–25 years, and the mean age of participants was 23 (± 2.2SD) with the age range of 18- 35 years [Table 1].

**Table 1:** Socio-demographic characteristics of the Study Participants with Symptomatic UTI ((*N* = 341) at Mekelle University, northern Ethiopia, February–September 2017

Variables	Frequency	Percent (%)
Sex		
Male	97	28.4
Female	244	71.6
Age (in years)		
16–19	29	8.5
21–25	217	63.6
26–30	90	26.4
31–35	5	1.5
Stay at Mekelle University (in years)		
1st	60	17.6
2nd	77	22.6
3rd	91	26.7
4th	73	21.4
5th and above	40	11.7
Residence (Campuses)		
Arid (Main Campus)	206	60.4
Adi Haqi	89	26.1
Ayder	34	9.9
Aynalam	6	1.8
Kalamino	6	1.8

### Prevalence of urinary tract infection

Of the 341 urine specimens analyzed, 72(21.1%) had significant bacteriuria (≥ 10^5^cfu/mL). The highest significant bacteriuria (23.3%) was observed in participants with the age group of 26–30 years **[**Table 2**].**

**Table 2:** Prevalence of UTI with Regard to the Socio-Demographic Characteristics among Mekelle UniversityStudents with Symptomatic UTI, northern Ethiopia, February–September 2017

Variables	Significant Bacteriuria		Percentage	Total
	Yes (%)	No (%)		
Sex				
Male	8(8.2)	89(91.8)	28.4	97
Female	64(26.2)	180(73.8)	71.6	244
Age (in years)				
16–20	5(17.2)	24(82.8)	8.5	29
21–25	46(21.2)	171(78.8)	63.6	217
26–30	21(23.3)	69(76.7)	26.4	90
31–35	0(0.0)	5(100.0)	1.5	5
Residence (Campuses)				
Arid (Main Campus)	39(19)	167(81)	60.4	206
Adi Haqi	21(23.4)	68	26.1	89
Ayder	11(32.4	23(67.6)	9.9	34
Aynalam	0(0.0)	6(100)	1.8	6
Kalamino	1(16.7)	5(83.3)	1.8	6

### Bacterial uropathogens

**Fig. 1:**
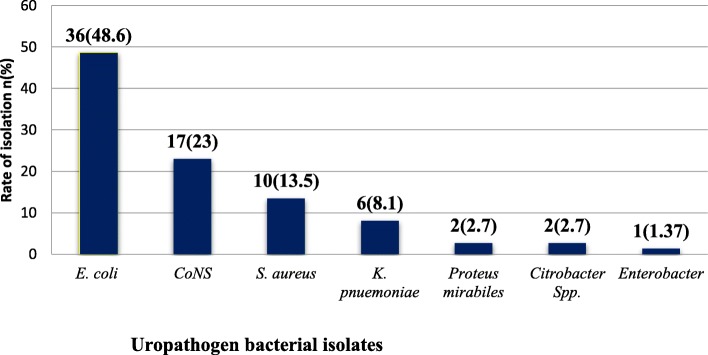
Frequency of bacterial uropathogen isolates among Mekelle University Studentswith symptomatic UTI, northern Ethiopia, February–September 2017

Of the total 74 isolates, 47(63.5%) were Gram-negative while 27(36.5%) were Gram-positive bacteria. Overall, *E.coli* was the dominant bacterial isolates 36(48.6%) followed by Coagulase-negative staphylococci (CoNS) 17(23%), *S. aureus* 10(13.5%) and *K.pneumonie* 6(8.1%). Of all, mixed pathogens were isolated from 2(2.8%) patients **[**Fig. 1**].**

### Antimicrobial susceptibility pattern of bacterial uropathogens

#### Gram negative bacteria

Gram negative isolates were resistant to ampicillin (100%), amoxicillin/clavulanic acid(93.6%), co-trimexazole(72.3%), nalidixic acid (57.4%), tetracycline (46.8%), gentamicin (29.8%) and chloramphenicol (25.5%).Antibiotics such as ciprofloxacin (85.1%), ceftriaxone (83.0%), norfloxacin (80.8%) and nitrofurantoin (78.7%) were more effective for the isolates **[**Table 3**].**

**Table 3:** Antimicrobial Susceptibility Pattern of Gram Negative Bacterial Isolates among Mekelle University Students with Symptomatic UTI, northern Ethiopia, February–September 2017

Bacterial Isolates(n)		Antibiotics										
		AMP	AMC	CTR	NX	GEN	NA	NIT	CIP	COT	TE	C
*E. coli* (36)	S	0(0.0)	2(5.6)	29(80.6)	30(83.3)	25(69.5)	15(41.7)	29(80.6)	30(83.3)	11(30.6)	21(58.3)	29(80.6)
	I	3(8.3)	3(8.3)	0(0.0)	2(5.6)	8(22.2)	5(13.9)	2(5.6)	3(8.3)	3(8.3)	3(8.3)	0(0.0)
	R	33(91.7)	31(86.1)	7(19.4)	4(11.1)	3(8.3)	16(44.4)	5(13.8)	3(8.3)	22(61.1)	12(33.3)	7(19.4)
*K. pneumoniae* (6)	S	0(0.0)	1(16.7)	5(83.3)	4(66.7)	4(66.7)	3(50.0)	3(50.0)	5(83.3)	1(16.7)	1(16.7)	3(50.0)
	I	1(16.7)	1(16.7)	0(0.0)	1(16.7)	0(0.0)	1(16.7)	0(0.0)	1(16.7)	1(16.7)	2(33.3)	2(33.3)
	R	5(83.3)	4(66.7)	1(16.7)	1(16.7)	2(33.3)	2(33.3)	3(50.0)	0(0.0)	4(66.7)	3(50.0)	1(16.7)
*P. mirabilis* (2)	S	0(0.0)	0(0.0)	2(100)	1(50.0)	2(100)	1(50.0)	2(100)	2(100)	1(50.0)	1(50.0)	2(100)
	I	0(0.0)	1(50.0)	0(0.0)	0(0.0)	0(0.0)	0(0.0)	0(0.0)	0(0.0)	0(0.0)	0(0.0)	0(0.0)
	R	2(100)	1(50.0)	0(0.0)	1(50.0)	0(0.0)	1(50.0)	0(0.0)	0(0.0)	1(50.0)	1(50.0)	0(0.0)
*Citrobacter spp.* (2)	S	0(0.0)	0(0.0)	2(100)	2(100)	1(50.0)	1(50.0)	2(100)	2(100)	0(0.0)	1(50.0)	1(50.0)
	I	0(0.0)	0(0.0)	0(0.0)	0(0.0)	1(50.0)	0(0.0)	0(0.0)	0(0.0)	0(0.0)	1(50.0)	0(0.0)
	R	2(100)	2(100)	0(0.0)	0(0.0)	0(0.0)	1(50.0)	0(0.0)	0(0.0)	2(100)	0(0.0)	1(50.0)
*Enterobacter spp*.(1)	S	0(0.0)	0(0.0)	1(100)	1(100)	1(100)	0(0.0)	1(100)	1(100)	0(0.0)	1(100)	0(0.0)
	I	0(0.0)	0(0.0)	0(0.0)	0(0.0)	0(0.0)	1(100)	0(0.0)	0(0.0)	0(0.0)	0(0.0)	1(100)
	R	1(100)	1(100)	0(0.0)	0(0.0)	0(0.0)	0(0.0)	0(0.0)	0(0.0)	1(0.0)	0(0.0)	0(0.0)
Total *n* (%)	S	0(0.0)	3(6.4)	39(83.0)	38(80.8)	33(70.2)	20(42.6)	37(78.7)	40(85.1)	13(27.7)	25(53.1)	35(74.5)
	I	4(8.5)	5(10.7)	0(0.0)	3(6.4)	9(19..2)	7(14.8)	2(4.3)	4(8.5)	4(8.5)	6(12.8)	3(6.4)
	R	43(91.5)	39(82.9)	8(17.0)	6(12.8)	5(10.6)	20(42.6)	8(17.0)	3(6.4)	30(63.8)	16(34.0)	9(19.1)

Key; *AMC* Amoxacillin clavulanic acid, *AMP* Ampicillin, *NA* Nalidixic acid, *NX* Norfloxacilin, *NIT* Nitrofruinton, *CTR* Ceftriaxone, *GEN* Gentamicin, *COT* Co-trimexazole, *TE* Tetracycline, *C* Chloramphenicol, *CIP* Ciprofloxacin, *S* Susceptible, *I* Intermediate, *R* Resistant. % is computed from each cell. *Total* total resistance of gram negative isolates for the specific tested

#### Gram-positive bacteria

Out of the tested antibiotics, Gram**-**positive bacterial isolates were highly resistant to ampicillin (81.5%), amoxicillin/clavulanic acid (77.8%), co**-**trimoxazole (55.6%) and tetracycline (55.5%). Among the tested antibiotics, ciprofloxacin (88.9%), gentamicin (85.2%), chloramphenicol (81.5%), nitrofurantoin (81.5%), ceftriaxone and erythromycin (74.1% each), and norfloxacin (70.4%) were effective to Gram**-**positive isolates **[**Table 4**].**

**Table 4:** Antimicrobial Susceptibility Patterns of Gram Positive Bacterial Isolates among Mekelle University Students with Symptomatic UTI, Northern Ethiopia, February–September 2017

Bacterial isolate(n)		Antibiotics										
		AMP	AMC	CTR	NX	GEN	NIT	CIP	COT	TE	ERY	C
*CoNS* (17)	S	4(23.5)	5(29.4)	15(88.2)	12(70.6)	17(100)	16(94.1)	17(100)	10(58.8)	8(47.1)	9(52.9)	14(82.3)
	I	1(5.9)	1(5.9)	1(5.9)	2(11.8)	0(0.0)	1(5.9)	0(0.0)	1(5.9)	6(35.3)	2(11.8)	1(5.9)
	R	12(70.6)	11(64.7)	1(5.9)	3(17.6)	0(0.0)	0(0.0)	0(0.0)	6(35.3)	3(17.6)	6(35.3)	2(11.8)
*S.aureus* (10)	S	1(10.0)	1(10.0)	6(60.0)	7(70.0)	6(60.0)	6(60.0)	7(70.0)	2(20.0)	4(40.0)	5(50.0)	4(80.0)
	I	0(0.0)	1(10.0)	1(10.0)	0(0.0)	1(10.0)	0(0.0)	0(0.0)	1(10.0)	0(0.0)	0(0.0)	0(0.0)
	R	9(90.0)	8(80.0)	3(30.0)	3(30.0)	3(30.0)	4(40.0)	3(30.0)	7(70.0)	6(60.0)c	5(50.0)	6(60.0)
Total *n* (%)	S	5(18.5)	6(22.2)	21(77.8)	19(70.4)	23(85.2)	22(81.5)	24(88.9)	12(44.4)	12(44.4)	14(51.9)	18(66.6)
	I	1(3.7)	2(7.4)	2(7.4)	2(7.4)	1(3.7)	1(3.7)	0(0.0)	2(7.4)	6(22.2)	2(7.4)	1(3.7)
	R	21(77.8)	19(70.)	4(14.8)	6(22.2)	3(11.1)	4(14.8)	3(11.1)	13(48.2)	9(33.3)	11(40.7)	8(29.6)

Key; *AMC* Amoxacillin clavulanic acid, *AMP* Ampicillin, *NX* Norfloxacilin, *NIT* Nitrofruinton, *CTR* Ceftriaxone, *GEN* Gentamicin, *COT* Co-trimexazole, *TE* Tetracycline, *ERY* Erythromycin, *C* Chloramphenicol, *CIP* Ciprofloxacin, *S* Susceptible, *I* Intermediate, *R* Resistant. % is computed from each cell, *Total* total resistance of gram positive isolates for the specific tested

#### Multiple drug resistance (MDR) patterns of the isolates

Among the total bacterial uropathogens isolates (*N* = 72), the overall prevalence of MDR in the present study was 54(73%). The prevalence of MDR for Gram-negative and positive isolates were 41(87.2%) and 13(48.1%), respectively [Table 5].

**Table 5:** Level of Antimicrobial Resistance of Uropathogen Bacterial Isolates among Mekelle University Students with Symptomatic UTI, Northern Ethiopia, February–September 2017

Bacterial isolates(n)	Antibiotics							
	Ro (%)	R1 (%)	R2 (%)	R3 (%)	R4 (%)	MDR (%)	ESBL producers(%)	Total (%)
Gram Negative	1(2.1)	1(2.1)	5(10.6)	20(42.6)	20(42.6)	41(87.2%)	12(25.5)	47(100)
*E.coli*	0(0.0)	1(2.8)	2(5.6)	18(50)	15(41.6)	33 (88.9)	10(27.8)	36(100)
*K. pneumonia*	1(16.7)	0(0.0)	1(16.7)	2(33.3)	2(33.3)	5(83.3)	2(33.3)	6(100)
*Citrobacter spp.*	0(0.0)	0(0.0)	1(50)	0(0.0)	1(50)	1(50)	0(0.0)	2(100)
*P.mirabilis.*	0(0.0)	0(0.0)	1(50)	0(.00)	1(50)	1(50)	0(0.0)	2(100)
*Enterobacter spps*.	0(0.0)	0(.00)	0(.00)	0(0.0)	1(100)	1(100)	0(0.0)	1(100)
Gram positive	5(18.5)	4(14.8)	4(18.5)	8(29.6)	6(18.5)	13(48.1%)	NT	27(100)
CoNS	4(23.5)	2(11.8)	3(17.6)	5(29.5)	3(17.6)	7(41.2)	NT	17(100)
*S. aureus*	1(10)	2(20)	1(10)	3(30)	3(30)	6(60)	NT	10(100)
Total	6(8.1)	5 (6.8)	9(12.2)	28(37.8)	26(35.1)	54(73.0)	12(25.5)	74(100)

Key;R0- susceptible to allantibiotics, R1- Resistance to one antibiotic, R2-Resistance to antibiotics, R3-Resistance to threeantibiotics, ≥ R4-resistance to four and above antibiotics, *NT* not test, *MDR* Multidrug resistant: non-susceptible to at least one agent in three antimicrobial categories [65]. Based on this definition, the following antimicrobial categories were considered to determine whether the given isolate is *MDR* Gram positive isolates and Enterobacteriacecae: aminoglycosides (gentamicin), cephems (ceftiraxone), pencillin (ampcillin), beta lactamase inhibitor combination (amoxicillin/clavulanic acid), fluoroquinolones (ciprofloxacin,norfloxacin), nitrofuran (nitrofurantoin), cotrimoxazole, tetracycline and phenicols (chloramphenicol). Of these, nalidixic acid and macrolides (erythromycin) was used only for *enterobacteriacecae* and Gram positive isolates

#### Extended-spectrum beta-lactamase (ESBL) production

Of the 47 Gram**-**negative isolates, 12(25.5%) were positive to ESBL production with10 (27.8%) of the *E.coli* isolates and 2(33.3%) of *K.pnuemoniae* **.**

#### Factors associated with bacterial urinary tract infections

Bivariate and multivariate logistic regression analyses were performed to assess the association between dependent and independent study variables. According to the bivariate analysis, being female, previous history of UTI, sexual activity, history of catheterization, uncircumcised male, contraceptive user females, Dysuria, Frequency, Urgency, Hematuria, Flank pain, and Urinary incontinence were showed association with symptomatic urinary tract infection and transported to multivariate analysis. Accordingly, in multivariate analysis, being female (AOR = 7.42, 95% CI: 1.25–43.96, *p* = 0.027), previous history of UTI (AOR = 5.75, 95%, CI: 1.70–19.4, *p* = 0.005), sexual activity (AOR = 2.16, 95% CI: 1.149–3.69, *p* = 0.012) and history of catheterization (AOR = 18.2, 95% CI: 4.45–74.77, *p* ≤ 0.001) were significantly factors associated with urinary tract infections. Among the currently observed symptoms of UTI, flank pain (AOR = 3.776, 95% CI: 1.55–9.18, *p* = 0.003) was significantly associated with urinary tract infections [Tables 6 and 7].

**Table 6:** Risk factors associated with UTI among Mekelle University students with symptomatic UTI, northern Ethiopia, February–September 2017

Variable		Total	Significant Bacteriuria		COR (95% CI)	*p*-value	AOR (95% CI)	*p*-value
			Yes (%)	No (%)				
Sex								
Male		97	8(8.2)	89(91.8)	1			
Female		244	64(26.2)	180(73.8)	3.96(2.45–14.02)	0.001	7.42(1.25–43.96)	0.027*
Age (in years)								
16–20		29	5(17.2)	24(82.8)	1			
21–25		217	46(21.2)	171(78.8)	1.29(0.242–3.26)	0.752	NA	
26–30		90	21(23.3)	69(76.7)	1.46(0.41–6.24)	0.505	NA	
31–35		5	0(0.0)	5(100.0)	NA			
Current symptoms								
Dysuria	No	91	9	82	1			
	Yes	250	63	187	3.06(1.263–5.632)	0.010	3.38 (1.001–11.44	0.052
Frequency	No	96	13	83	1			
	Yes	245	59	186	2.02(1.004–3.593)	0.048	0.73 (0.279–1.93)	0.534
Urgency	No	110	14	103	1			
	Yes	231	58	173	2.46 (1.567–5.719)	0.001	1.605(.637–4.046)	0.316
Hematuria	No	290	51	239	1			
	Yes	51	21	30	3.28 (1.370–6.34)	0.006	0.812(0.26–2.58)	0.725
Abdominal pain	No	80	13	67	1			
	Yes	261	59	202	1.50(.813–3.040)	0.179	NA	
Fever	No	147	30	117	1			
	Yes	194	42	152	1.07 (.633–1.795)	0.811	NA	
Flank pain	No	248	39	209	1			
	Yes	93	33	60	2.95 (2.12–10.955)	0.001	3.776(1.552–9.18)	0.003*
Urinary Incontinence	No	215	29	186	1			
	Yes	126	43	83	3.32 (3.007–9.116)	0.001	2.07 (0.855–4.99)	0.107

**Table 7:** Association of Other Enabling Factors with UTI among Mekelle University Students with Symptomatic UTI, Northern Ethiopia, February–September 2017

Variable	Total	Significant Bacteriuria		COR (95%,CI)	*p*-value	AOR (95%,CI)	*p*-value
		Yes	No				
History of UTI							
No	299	46	253	1			
Yes	42	26	16	8.93 (5.27–28.14)	0.001	5.75 (1.70–19.4)	0.005*
History of DM							
No	316	66	250	1			
Yes	15	6	9	2.52 (.130–12.411)	0.836	NA	
Using contraceptive							
No	313	60	253	1			
Yes	28	12	16	3.16(2.30–8.427)	0.001	1.14(0.412–3.17)	0.798
History of Medication							
No	297	57	240	1			
Yes	44	15	29	2.17(1.53–14.46)	0.001	1.98(0.422–9.27)	0.386
History of Catheter							
No	330	66	264	1			
Yes	11	6	5	4.8 (1.68–22.387)	0.006	18.2 (4.45–74.77)	0.001*
Male Circumcision							
No	19	3	16	2.73 (2.120–14.063)	0.001	3.89(0.66–71.56)	0.106
Yes	78	5	73	1			
Female Sexual Activity							
Never had Sex	177	43	134	1			
Had sex < 3 per week	61	16	45	1.1(1.004–3.593)	0.048	1.605(0.637–4.05)	0.316
Had sex ≥3 per week	11	5	6	2.59 (1.26–6.12)	0.015	2.16 (1.149–3.69)	0.012*

Note: *Statistically significant at *P* < 0.05.DM, diabetes mellitus; *AOR* adjusted odds ratio, *COR* crude odds ratio, 1 referent group, *CI* confidence interval, *N/A* not applicable

## Discussion

Urinary tract infection (UTI) remains to be one of the most common infectious diseases diagnosed in the community [33, 34].

The overall prevalence of UTI in this study was found to be 21.1%; which was in agreement with the findings of the previous studies conducted in Ethiopia from the general population (23.32%) [35]; Keffi, Nigeria (20%) [36] and Ogun State, Nigeria (25%) [37]; India (19.8%) [13] and (22%) [38]. However, our finding was higher than other earlier studies reported in Southeast Nigeria (13.8%) [39]; and BeninCity, Nigeria (11%) [40] and (8.25%) [41]. But it was lower compared to other studies done in Nigeria, Imo State University (28%) [20] southeastern Nigeria (78%) [42]. This difference in the rate of UTI may be explained by variation in the methodology used, sexual behavior (those sexually active individuals are more exposed to urinary tract infection). This is due to ascending infection from genital to the urinary tract, climatic and geographic variation might be attributed to cold climate that leads to alack of personal and environmental hygiene of participants, lack of sanitary materials in the university such as access of water, low socioeconomic status similar to the previous finding in Iran [3], majority of the isolates (89.2%) were from female participants which support the implication of females are at high risk for UTI [10, 11]. This high prevalence of UTI among female participants may be due to females have shorter and wider urethra which is proximate to the anus, lack of prostatic fluid which acts as an antimicrobial agent; and having warm and moist urethra which could be supportive for the optimal growth of bacteria compared to males [43]. Also besides, other behavioral factors such as the mechanical introduction of pathogens into the bladder and trauma effect during sexual intercourse could also be a reason for this high prevalence of UTI among female individuals [44].

As studies have been documented in the general populations the etiologic agents of UTI mostly belong to Gram**-**negative bacteria [15, 21]. Likewise,63.5% isolates of the present study were Gram-negative bacteria. Of all, *E. coli* was the most frequent bacterial isolate with a 48.6% isolation rate. This isolation rate of *E. coli* was similar to earlier findings reported in other parts of Ethiopia (47.5%) [45] and (44.62%) [37]; and Sudan (47.3%) [46]. The possible explanation for this high isolation rate of *E. coli* in the present finding could be due to the contamination of the urinary tract from the rectal area and it could also be due to *E. coli* has various enhanced virulence factors specific for colonization and invasion of the urinary epithelium [35]. The second most common isolate was CoNS(23.0%). This was in line with findings reported from the general population in Ethiopia (22.5%) [46] and (22%) [47]. This could be as CoNS are a normal flora of the urogenital area at puberty, which may invade the urinary tract during sexual activity especiallyinfemales [1]. Isolation rate of *S.aureus* (13.5%) and *K.pneumoniae* (.1%) in our study was comparable to previous reports in Nigeria (13.3%) and (6.4%)respectively [37]. Similar to previously conducted studies in Ethiopia [47], (2.8%) of mixed bacterial pathogens were isolated in the present study.

The present study showed participants with a past history of UTI had significantly higher prevalence UTI compared to those with no previous history UTI (*P* = 0.005). This was similar to findings reported in Ethiopia [29, 47] and another place [11]. The possible explanation for this association could be due to the existence of resistant strains from the earlier uropathogens.

In line with previously documented results [1, 3, 11] our study revealed, female participants had seven folded increased risk of acquiring UTI. This might be due to females have short, wider and direct urethra, lack of prostatic fluid which acts as antimicrobial; and having warm and moist urethra which could be supportive for the optimal growth of bacteria compared to males [37]. The history of catheterization was also found significantly associated with the presence of UTI in this study (*p* < 0.001). This was similar to a study done in Ethiopia [43]. This could be due to contamination while catheter insertion, frequent and long-term catheterization which supports adherence of pathogens to the urinary tract. Sexual activity was also the other risk factor that was found to be statistically associated with UTI. Females who had recent sexual intercourse of three or more per week were two times more likely to have UTI than females who had less than three intercourses per week. This was in line with the result of the previous study in Ethiopia [29]. The possible explanation for this association could be due to the frequent use of contraceptives in addition to having frequent intercourse may push the bacteria into the bladder as explained above [29].

In line with earlier documented findings [2, 39, 48]; age and history of antibiotics had no association with UTI in the present study. Moreover, contrary to the previous report [46] the present study revealed, using contraceptives, circumcision and having diabetics’ Mellitus had no significant association with the presence of UTI.

Our study revealed Gram**-**negative isolates were 100%resistant to ampicillin. This rate of resistance was similar to the previous study done in Ethiopia [45] and Nigeria [39] who reported (100%).In contrast to the previous documented resistant rate of Gram**-**negative bacteria to amoxicillin/clavulanic acid in Ethiopia (30%) [45], and (40.7%) [35], a higher rate of resistance was observed in our study (93.6%). This resistance rate was similar to a report from Nigeria (96.5%) [29]. This high resistance rate of Gram-negative isolates against amoxicillin/clavulanic acid could be due to the production of inhibitors resistant β-lactamase. The resistance rate of Gram**-**negative isolates to co**-**trimoxazole and nalidixic acid was (72.3%) and (57.4%) respectively. These rates of resistance were comparable to previous reports from Uganda co-trimoxazol (80%) [49]; India, nalidixic acid (51%) [50]. The factors contributing to those resistance rates might be due to the irrational use /self-medication of antibiotics in the study area that is common [8]. However, the resistance rate of Gram**-**negative isolates to tetracycline (46.8%) in the present study was lower compared to the previous report in Ethiopia (76.9%) [37]. generally, most Gram**-**negative isolates of the present study were sensitive to ciprofloxacin (85.1%), ceftriaxone (83.0%), norfloxacin (80.8%), nitrofurantoin (78.7%) and chloramphenicol (74.5%). This finding was in line with previous findings from Bangladesh [34] and Nigeria [39] where ciprofloxacin, ceftriaxone, norfloxacin, nitrofurantoin, and chloramphenicol were effective to Gram**-**negative isolates.

Among Gram-positive isolates *S. aureus* showed a high resistance rate to ampicillin and amoxicillin/clavulanic acid (90% each), co**-**trimoxizole (80%) followed by tetracycline (60%) and erythromycin (50%). This high trend of resistance was comparable with earlier documented results in Ethiopia, ampicillin (87.5–100%) [48] and tetracycline (57.1%) [2]. This higher resistance rate of *S. aureus* could be due to the production of penicillinase enzymes and other alternative penicillin**-**binding proteins which helps the organism to become resistant to β-lactam antibiotics in addition to the other resistance mechanisms and irrational use of these antibiotics. Generally, gram**-**positive isolates of the present study were highly resistant to ampicillin(81.5%), amoxicillin/clavulanic acid (77.8%), co-trimexazole(55.6%), and erythromycin(48.2%). This resistance rate was a bit lower compared to finding reported in Nigeria, ampicillin (88.9%) and tetracycline (66.7%) [39]. It was, however, comparable with the previously documented results in Ethiopia which resistance rate to tetracycline and co**-**trimexazole was around (50%) [21]. On the contrary, the resistance rate of Gram**-**positive isolates to erythromycin and amoxicillin/clavulanic acid of this study was higher than an earlier study conducted in Ethiopia which showed resistance rate to erythromycin (25%) and amoxicillin/clavulanic acid (0%) [21]. This might be due to inappropriate use and incorrect administration of these antibiotics in addition to other factors like strain and geographic variation.

The overall prevalence of MDR in this study (73%) was comparable with the previous finding in Nigeria (81.4%) [28]. However, our result was lower than earlier reported prevalence of MDR from Ethiopia (95–100%) [2, 45]. In contrast, the result of this study was higher than other documented results in Ethiopia (59.8%) [47]. The high prevalence of MDR in the present study could also be due to multiple resistant genes that can develop on the mobile genetic elements [51] and plasmids bearing genes-encoding ESBLs, frequently also carry genes encoding resistance to other antimicrobial agents [52]. Self-medication which is a common practice in the study area [8] also might have a great role in the development of such a higher prevalence of MDR [28].

The treatment choice of ESBL-producing organisms is very restricted [52]. Similar to a previously reported article in western India (21.3%) [50], the overall ESBL production of Gram**-**negative isolates of the present study was 25.5%. ESBL production of *Klebsiella* isolates (33.3%) in the present study was similar to previous findings in Ethiopia (33.3%) [53]. However, the overall ESBL production of this study was lower compared to finding reported in Ethiopia (78.57%) [54] and Nigeria, 34.9% [29]. This high ESBL production of isolates might be due to uncontrolled antibiotic usage, inappropriate dosing regimens and substandard antibiotics which are a risk factor for the acquisition of an ESBL-producing organism [52].

## Conclusion

In the present study, the overall prevalence of UTI was 21.1%. Being female, the previous history of catheterization, prior history of UTI and frequent sexual activity had a statistically significant association with the occurrence of UTI.*E. coli* was the most dominant isolate followed by CoNS and most of the isolates were highly resistant to ampicillin, amoxicillin/clavulanic acid and co-trimoxazole followed by nalidixic acid and tetracycline. MDR was seen in 73% of the isolates and25.5% of the Gram**-**negative bacteria were ESBL producers. Safe sexual intercourse, safe catheterization should be applied to lessen the magnitude UTI. Therefore, from this study, there is a significant increase in UTI and antibiotic resistance in university students. Our study suggests early diagnosis and initiation antibiotic for UTI and antimicrobial susceptibility tests should be recommended to prevent serious complications.

## Data Availability

The datasets used and analyzed during the current study are available from the corresponding author on reasonable request.

## Abbreviations

ATCC: American Type Culture Collection
CFU: Colony Forming Unit
CLSI: Clinical and Laboratory Standards Institute
CoNS: Coagulase**-**negative staphylococci
DM: Diabetes Mellitus
ESBL: Extended-Spectrum Beta**-**Lactamase
MSU: Midstream Urine
MU: Mekelle University
SB: Significant Bacteriuria
SD: Standard Deviation
SPSS: Statistical Package for Social Science
TSI: Triple Sugar Iron Agar
US: United States
UTI: Urinary Tract Infection
WHO: World Health Organization
